# TGF-β-SNAIL axis induces Müller glial-mesenchymal transition in the pathogenesis of idiopathic epiretinal membrane

**DOI:** 10.1038/s41598-018-36917-9

**Published:** 2019-01-24

**Authors:** Atsuhiro Kanda, Kousuke Noda, Ikuyo Hirose, Susumu Ishida

**Affiliations:** Laboratory of Ocular Cell Biology and Visual Science, Department of Ophthalmology, Faculty of Medicine and Graduate School of Medicine, Hokkaido University, Sapporo, Hokkaido, 060-8638 Japan

## Abstract

The epithelial-mesenchymal transition (EMT) is a key process in fibrogenic diseases where transdifferentiated myofibroblasts produce excessive amounts of extracellular matrix, resulting in organ dysfunction. Idiopathic epiretinal membrane (iERM) is a vision-threatening disorder characterized by fibrocellular proliferation and contraction on the central retina. Müller glial cells, which regulate retinal physiology and structure, are the major cellular components in the iERM tissue; however, the pathological role of this cell type remains incompletely understood. Here we revealed the involvement of Müller glial-mesenchymal transition (GMT), as an alternative to EMT, in the pathogenesis of iERM lacking epithelial contribution in nature. Of various pro-fibrotic cytokines, transforming growth factor (TGF)-β1 stimulation to human Müller glial cells exclusively increased mRNA and protein levels of several EMT-related molecular markers, together with the transcription factor SNAIL but not SLUG or TWIST. TGF-β1-stimulated Müller cells also exhibited EMT-related cell motility, while reducing the expression of glutamine synthetase (GS), a Müller glial marker. Notably, all of these TGF-β-induced EMT features were reversed by *SNAI1* knockdown in Müller cells. iERM patient specimens demonstrated co-immunolocalization of SNAIL with TGF-β1, GS, and smooth muscle protein 22. Our data implicated a critical role of the TGF-β-SNAIL axis in Müller GMT to promote iERM formation.

## Introduction

The epithelial-mesenchymal transition (EMT) is a complex biological process characterized by the transdifferentiation of epithelial cells into motile mesenchymal cells^1–4^. In addition to its physiological involvement in embryogenesis and organ morphogenesis (Type 1 EMT), the equivalent cellular system also applies to normal wound healing and repair as well as excessive tissue remodeling due to fibrogenesis (Type 2 EMT)^1^. The other detrimental diversion of the EMT program in terms of cell motility and growth contributes to tumor progression, invasion, and metastasis, thereby promoting carcinogenesis (Type 3 EMT)^1^. In Type 2 EMT-mediated tissue fibrosis, highly transdifferentiated myofibroblasts acquire the following pathogenic phenotypes: aberrant cell migration and proliferation, extracellular matrix (ECM) overproduction, and cytoskeletal muscle contraction; thus resulting in tissue deformation and organ dysfunction^1,5^. Although several pro-fibrotic cytokines including connective tissue growth factor (CTGF), fibroblast growth factor (FGF), and platelet-derived growth factor (PDGF) have been described, transforming growth factor (TGF)-β signaling via TGF-β receptor (TβR) is regarded as the major trigger of EMT and tissue fibrosis in various organs^1–5^. As concerns ocular fibrosis, TGF-β-induced EMT was shown to occur in retinal pigment epithelial (RPE) cells, a characteristic event seen in proliferative vitreoretinopathy and age-related macular degeneration, and also in lens epithelial cells, leading to anterior subcapsular cataract and posterior capsular opacification^5–9^. TGF-β-TβR downstream pathways induce the activation of several transcription factors integral to the execution of the EMT program, including SNAIL, SLUG, and TWIST, all of which can modify the expression of multiple genes so as to enhance myofibroblastic differentiation in a variety of epithelial cells^2–4^. The Type 2 EMT program would therefore be established on a basis of the essential combination of pro-fibrotic stimuli, transcription factors, and resultant cellular phenotypes, *i*.*e*., cell motility, ECM productivity, and cytoskeleton contractility.

Idiopathic epiretinal membrane (iERM) is a vision-threatening disorder characterized by fibrocellular proliferation and contraction on the surface of the retina at the macular region, *i*.*e*., the central area of the ocular fundus. The risk factors of iERM were shown to include aging with the peak prevalence rate (roughly 10–35%) between 70 and 79 years of age, as well as posterior vitreous detachment and cataract surgery, both of which are age-related^10^. During the formation of iERM, ECM proteins such as type I collagen are secreted in a sheet-like fashion by epiretinal cellular components with contractile activity via α-smooth muscle actin (SMA), which eventually shrink involving the underlying central retinal tissue and thus impairing visual acuity^10^. These fibrogenic processes closely correspond with those of Type 2 EMT featuring myofibroblasts, which are indeed one of the major cellular participants in the iERM tissue^10^; however, the origin of myofibroblasts remains largely unclear in the pathogenesis of iERM lacking epithelial contribution in nature. The major cellular source for myofibroblastic differentiation is suggested to be Müller glial cells whose presence and pro-fibrotic role in iERM have been demonstrated in several immunohistochemical and *in vitro* studies^11–13^. Moreover, Müller cells undergo reactive gliosis characterized by cell proliferation and cytoplasmic extension, both of which contribute to epiretinal scar formation^14,15^. However, the precise molecular mechanism causing fibrosis as well as myofibroblastic differentiation in Müller cells has yet to be elucidated in terms of whether the EMT program is appropriated to Müller glial cells of non-epithelial origin.

In this study, we investigated the possibility of Müller glial-mesenchymal transition (GMT), as an alternative to EMT, functioning as a driving force of iERM formation. To verify this, we checked the aforementioned parameters of the Type 2 EMT program by screening pro-fibrotic cytokines that transdifferentiate Müller cells into myofibroblasts, analyzing whether the transdifferentiated cells exhibit fibrogenic phenotypes (cell motility, ECM productivity, and cytoskeleton contractility), and determining which transcription factor governs these Type 2 EMT features in human Müller glial cells. These *in vitro* data were further supported by immunohistochemistry for iERM patient specimens.

## Results

### TGF-β1 and TGF-β2, but not other pro-fibrotic cytokines, exclusively induces the expression of EMT markers in Müller glial cells

**Figure 1:**
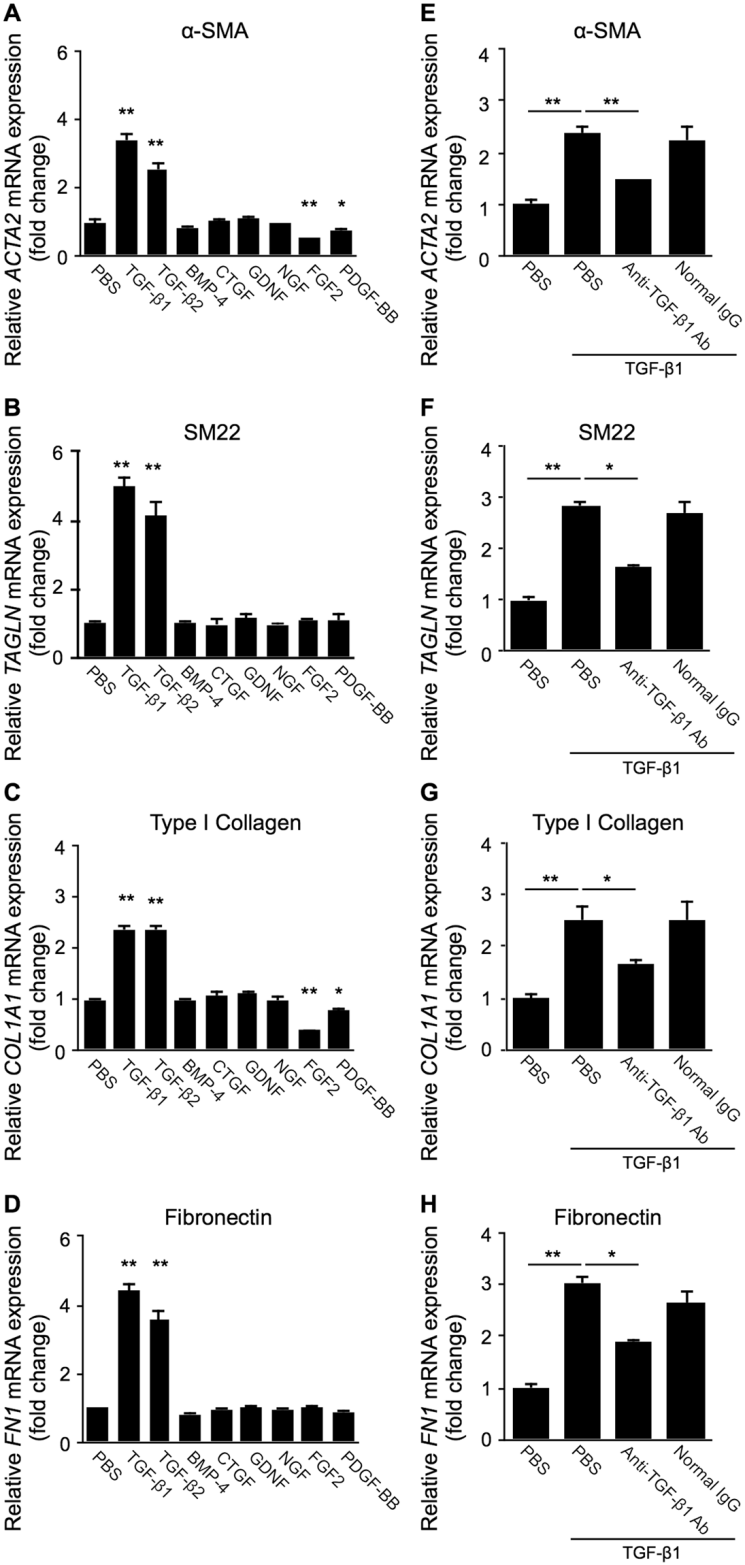
TGF-β1 and TGF-β2, but not other pro-fibrotic cytokines, exclusively induces the expression of EMT markers in Müller glial cells. (**A–D**) Müller glial cells were treated with TGF-β1, TGF-β2, BMP-4, CTGF, GDNF, NGF, FGF2, and PDGF-BB at the dose of 10 ng/ml for 24 hours, and *ACTA2* (**A**), *TAGLN* (**B**), *COL1A1* (**C**), and *FN1* (**D**) expression levels were analyzed. *E-H*, Müller glial cells were treated for 24 hours with the reaction mixture of 10 ng/ml TGF-β1 preincubated with anti-TGF-β1 neutralizing antibody or control normal IgG at the dose of 200 ng/ml for 15 minutes, and *ACTA2* (**E**), *TAGLN* (**F**), *COL1A1* (**G**), and *FN1* (**H**) gene expression levels were analyzed. n = 5–6 per group, *p < 0.05, **p < 0.01.

**Figure 2:**
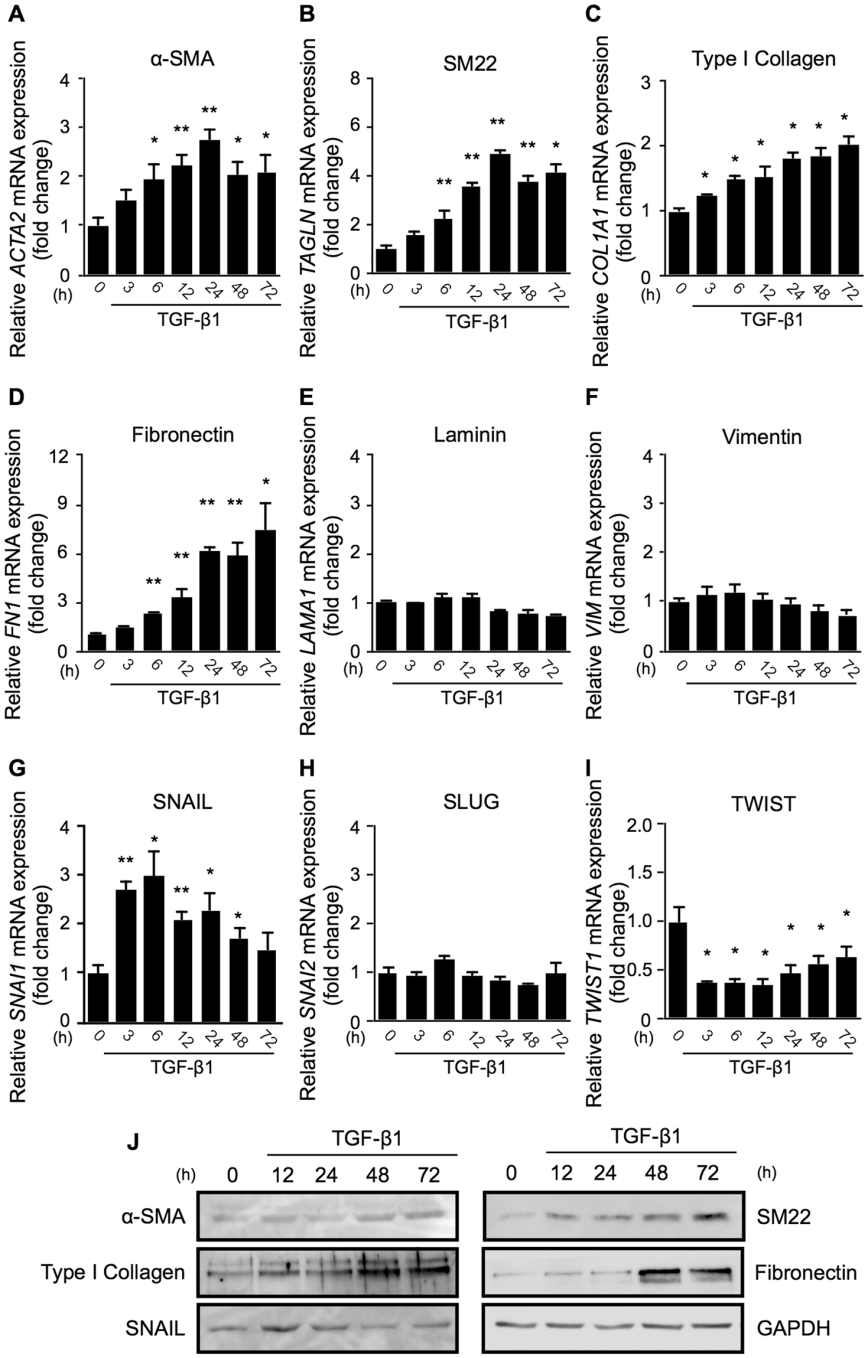
TGF-β1 upregulates SNAIL, but not SLUG or TWIST, followed by the expression of Type 2 EMT phenotypes in Müller glial cells. (**A–J**) Müller glial cells were treated with or without 10 ng/ml TGF-β1. (**A–I**) Real-time quantitative PCR analyses for the time course of expression levels of *ACTA2* (**A**), *TAGLN* (**B**), *COL1A1* (**C**), *FN1* (**D**), *LAMA1* (**E**), *VIM* (**F**), *SNAI1* (**G**), *SNAI2* (**H**), and *TWIST1* (**I**). h, hours. n = 6 per group, *p < 0.05, **p < 0.01. (**J**) Immunoblot analyses for the time course of protein expression of α-SMA, SM22, type I collagen, fibronectin, SNAIL, and GAPDH. Full size blots are shown in Supplementary Fig. S1.

**Figure 3:**
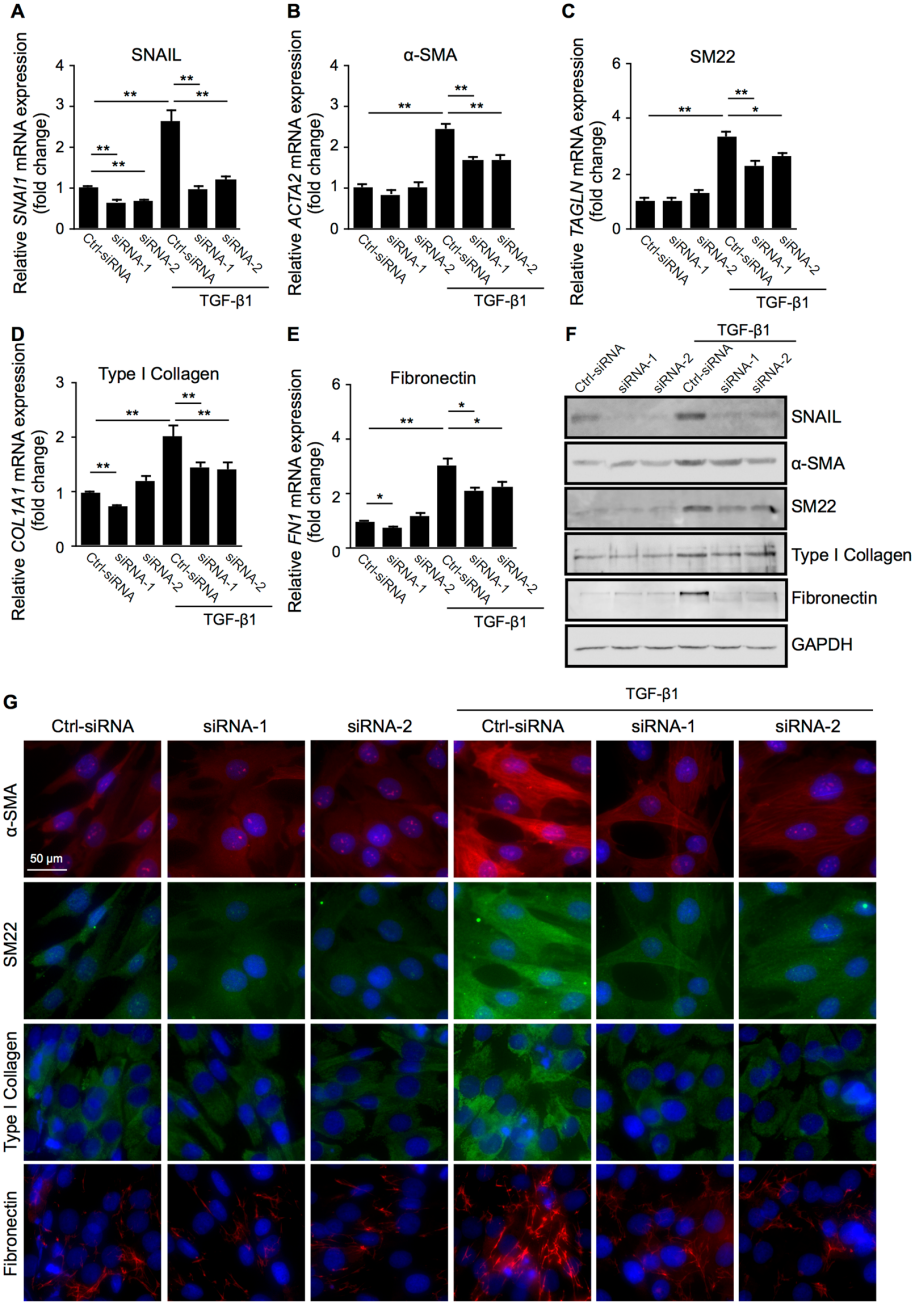
SNAIL is required for TGF-β1-induced expression of EMT-related molecular markers in Müller glial cells. (**A–G**) Control siRNA-treated (Ctrl-siRNA) and *SNAI1*-knockdown (siRNA-1 and -2) Müller glial cells were treated with or without 10 ng/ml TGF-β1 for 24 hours. (**A–E**) Real-time quantitative PCR analyses for *SNAI1* (**A**), *ACTA2* (**B**), *TAGLN* (**C**), *COL1A1* (**D**), and *FN1* (**E**). n = 6 per group, *p < 0.05, **p < 0.01. (**F**) Immunoblot analyses for SNAIL, α-SMA, SM22, type I collagen, fibronectin, and GAPDH. Full size blots are shown in Supplementary Fig. S2. (**G**) Immunocytochemical analyses for α-SMA, SM22, type I collagen, and fibronectin. Scale bar = 50 μm.

**Figure 4:**
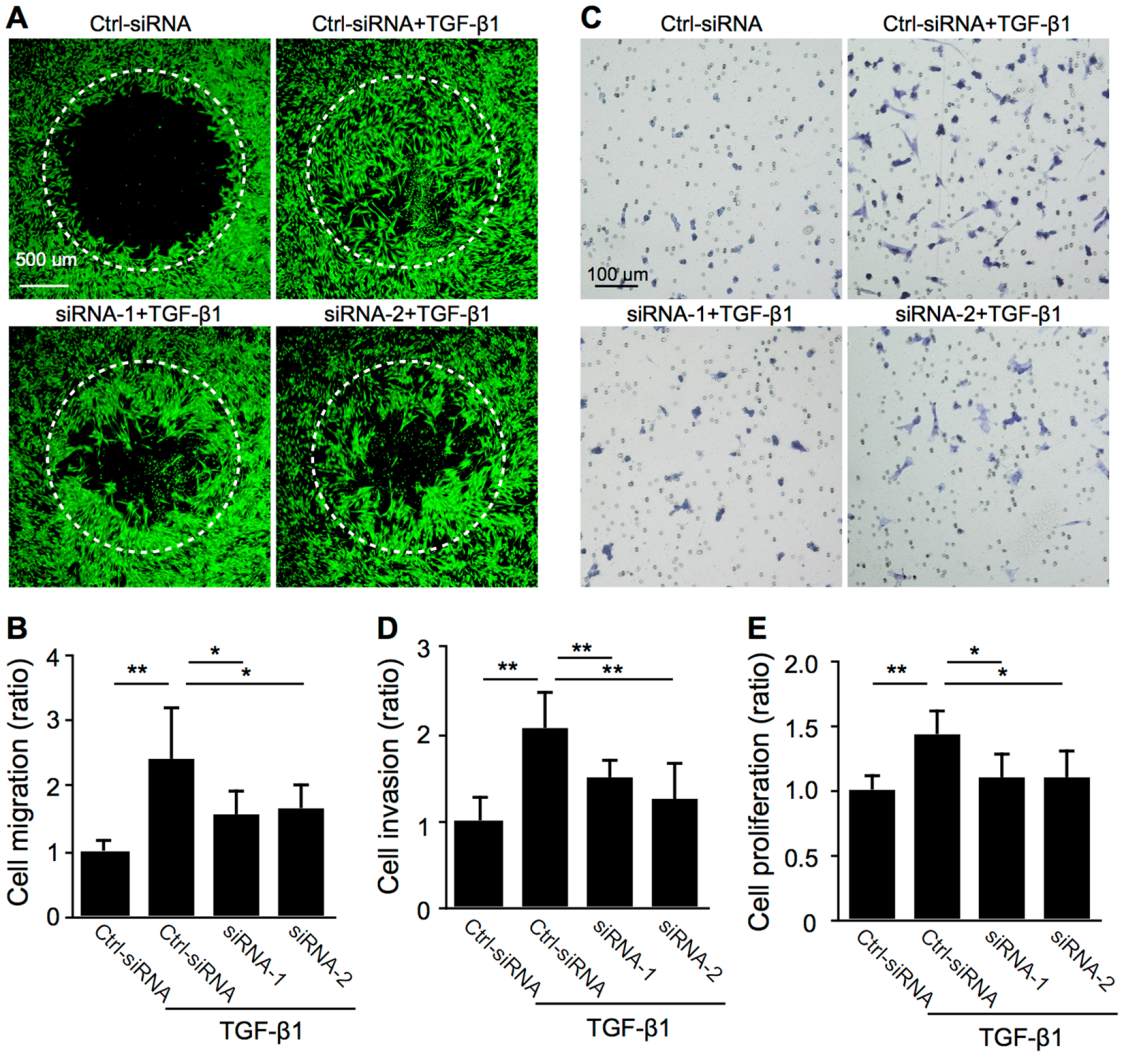
SNAIL is required for TGF-β1-induced cell motility in Müller glial cells. (**A–E**) Control siRNA-treated (Ctrl-siRNA) and *SNAI1*-knockdown (siRNA-1 and -2) Müller glial cells were stimulated with or without TGF-β1 at 10 ng/ml. (**A**,**B**) Cell migration assay. Representative images of cells stained with calcein AM. (**A**) The signal intensity of stained cells having migrated within the detection zones (white dotted circles) after 48 hours was measured (**B**). n = 7–10 per group. Scale bar = 500 μm. (**C**,**D**) Cell invasion assay. Representative images of cells stained with Toluidine Blue O (**C**). The number of stained cells having invaded on the bottom side of Matrigel-coated filter after 24 hours was counted (**D**). n = 3 per group. Scale bar = 100 μm. (**E**) Cell proliferation assay. The optical density for BrdU incorporation after 24 hours was measured. n = 8 per group, *p < 0.05, **p < 0.01.

**Figure 5:**
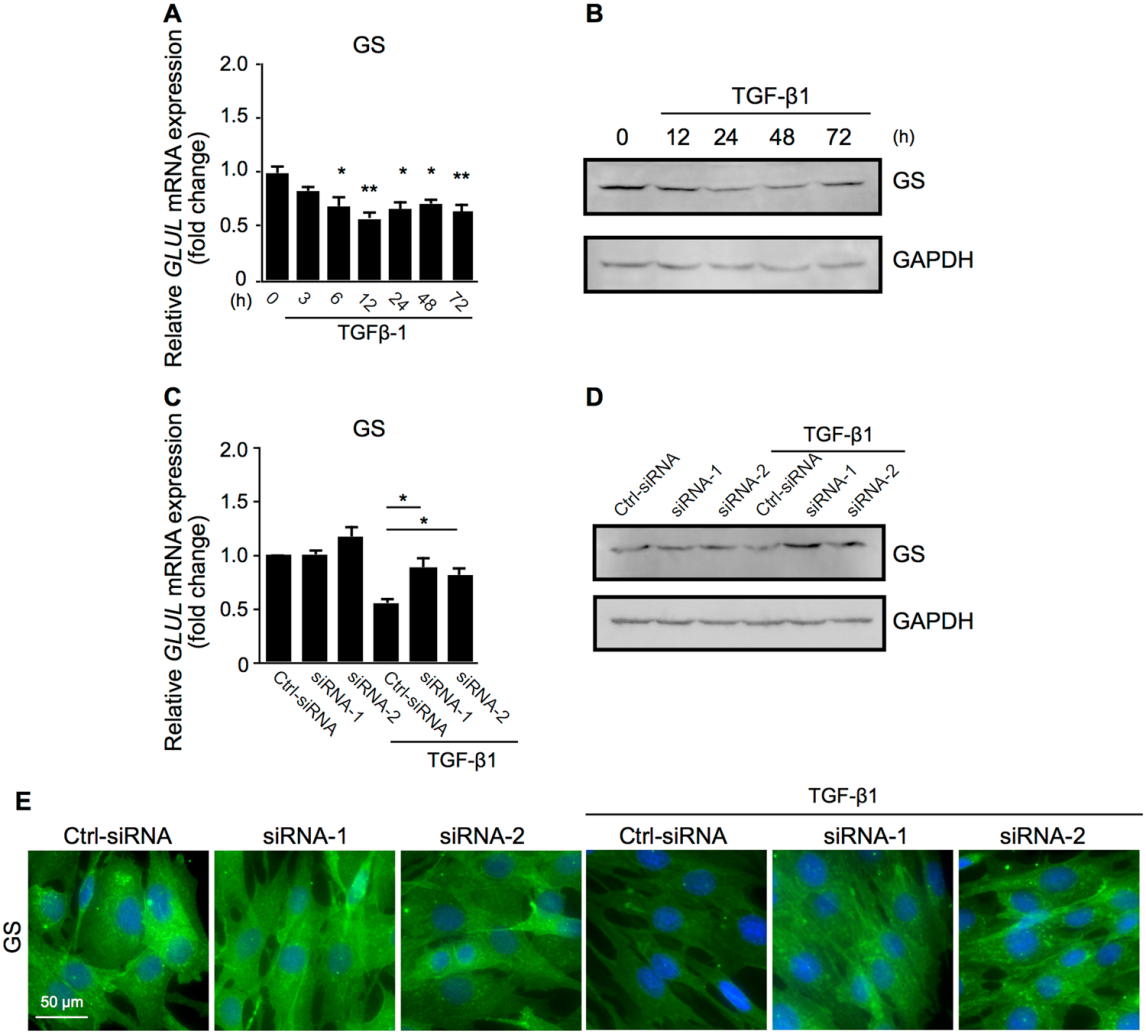
SNAIL is required for TGF-β1-induced loss of Müller glial property. (**A**,**B**) Müller glial cells were treated with or without 10 ng/ml TGF-β1. (**A**) Real-time quantitative PCR analyses for the time course of expression levels of *GLUL*. n = 6 per group, *p < 0.05, **p < 0.01. (**B**) Immunoblot analyses for the time course of protein expression of GS and GAPDH. h, hours. Full size blots are shown in Supplementary Fig. S3A. (**C–E**) Control siRNA-treated (Ctrl-siRNA) and *SNAI1*-knockdown (siRNA-1 and -2) Müller glial cells were treated with or without 10 ng/ml TGF-β1 for 24 hours. (**C**) Real-time quantitative PCR analyses for *GLUL*. n = 6 per group, *p < 0.05. (**D**) Immunoblot analyses for GS and GAPDH. Full size blots are shown in Supplementary Fig. S3B. (**E**) Immunocytochemical analyses for GS. Scale bar = 50 μm.

To investigate which pro-fibrotic cytokine can induce mesenchymal (EMT-like) changes in human Müller glial cells, we stimulated MIO-M1 cells with various cytokines and growth factors known for their fibrogenic activity and/or their protein expression in the iERM tissue^12,16,17^, and analyzed mRNA expression levels of several EMT-related molecular markers by real-time quantitative PCR. Smooth muscle protein (SM)22, also known as transgelin encoded by the *TAGLN* gene, is an actin-binding cytoskeletal protein recently utilized as another marker for myofibroblasts and mesenchymal cells, on top of conventionally used α-SMA^18–20^. Of various pro-fibrotic stimuli with TGF-β1/2, bone morphogenic protein (BMP)-4, CTGF, glial cell-derived neurotrophic factor (GDNF), nerve growth factor (NGF), FGF2, and PDGF-BB to Müller cells, TGF-β1 as well as TGF-β2 alone exclusively increased the gene expression of *ACTA2* (α-SMA) (fold change; TGF-β1 = 3.41, TGF-β2 = 2.53), *TAGLN* (SM22) (fold change; TGF-β1 = 5.01, TGF-β2 = 4.12), *COL1A1* (type I collagen) (fold change; TGF-β1 = 2.36, TGF-β2 = 2.37), and *FN1* (fibronectin) (fold change; TGF-β1 = 4.41, TGF-β2 = 3.59), as compared to PBS controls (Fig. 1A–D). Next, to strictly rule out the possibility of non-specific, TGF-β-unrelated response, we further performed antibody-based blocking experiments. Pretreatment with anti-TGF-β1 neutralizing antibody reversed TGF-β1-induced expression of these EMT markers (fold change; *ACTA2* = 1.46, *TAGLN* = 1.65, *COL1A1* = 1.68, *FN1* = 1.89), while normal IgG did not show any recovery (fold change; *ACTA2* = 2.25, *TAGLN* = 2.69, *COL1A1* = 2.52, *FN1* = 2.64) (Fig. 1E–H), substantially ensuring TGF-β1’s stimulatory bioaction on MIO-M1 cells. In the following *in vitro* assays (Figs 2–5), therefore, we chose to use TGF-β1 as a potential Type 2 EMT inducer in Müller glial cells.

### TGF-β1 upregulates SNAIL, but not SLUG or TWIST, followed by the expression of Type 2 EMT phenotypes in Müller glial cells

To examine whether TGF-β1 triggers the execution of the Type 2 EMT program in human Müller glial cells, we performed further experiments focusing on additional EMT markers including transcription factors, together with the time course of their expression. TGF-β1 application to MIO-M1 cells elevated the mRNA levels of *ACTA2* (fold change; 6 h = 1.95, 12 h = 2.22, 24 h = 2.76, 48 h = 2.03, 72 h = 2.07), *TAGLN* (fold change; 6 h = 2.24, 12 h = 3.56, 24 h = 4.91, 48 h = 3.75, 72 h = 4.12), *COL1A1* (fold change; 3 h = 1.24, 6 h = 1.48, 12 h = 1.52, 24 h = 1.82, 48 h = 1.83, 72 h = 2.03), and *FN1* (fold change; 6 h = 2.32, 12 h = 3.34, 24 h = 6.19, 48 h = 5.91, 72 h = 7.38) in a time-dependent manner (Fig. 2A–D), while no changes in *LAMA1* (laminin) or *VIM* (vimentin) gene expression were detected (Fig. 2E,F). As for TGF-β’s downstream transcription factors known to induce EMT in epithelial cells^2–4^, TGF-β1 stimulation to Müller glial cells significantly increased the expression levels of *SNAI1* (SNAIL) (fold change; 3 h = 2.71, 6 h = 2.98, 12 h = 2.09, 24 h = 2.29, 48 h = 1.72), while those of *SNAI2* (SLUG) and *TWIST1* (TWIST) were unaltered and reduced, respectively (Fig. 2G–I). Importantly, the duration required to reach maximal mRNA induction was much shorter in the transcription factor SNAIL (6 hours) than in the smooth muscle markers α-SMA and SM22 (24 hours for both) and in the ECM constituents type I collagen and fibronectin (72 hours or later for both). Moreover, immunoblot analyses confirmed the similar time course of changes in the earlier protein expression of SNAIL (peak at 12 hours) compared to these contractile cell proteins (peak at 72 hours or later) and ECM proteins (peak at 48 hours or later) (Fig. 2J), suggesting SNAIL as a candidate cue to initiate these Type 2 EMT changes in Müller glial cells.

### SNAIL is required for TGF-β1-induced expression of EMT-related molecular markers in Müller glial cells

To test a requirement of SNAIL for TGF-β1-induced expression of contractile cell markers (α-SMA and SM22) and ECM components (type I collagen and fibronectin) in Müller glial cells (Fig. 2A–D,J), *SNAI1* mRNA was knocked down via application of two different siRNAs (siRNA-1 and -2) to MIO-M1 cells. Both siRNAs significantly repressed the mRNA levels of *SNAI1* in non-stimulated cells (fold change; siRNA-1 = 0.66, siRNA-2 = 0.68) as well as in TGF-β1-stimulated cells (fold change; Ctrl-siRNA = 2.65, siRNA-1 = 0.99, siRNA-2 = 1.19) (Fig. 3A). Of note, the depletion of *SNAI1* prevented TGF-β1-induced upregulation of the EMT-related gene transcripts: *ACTA2* (fold change with TGF-β1; Ctrl-siRNA = 2.42, siRNA-1 = 1.67, siRNA-2 = 1.68), *TAGLN* (fold change with TGF-β1; Ctrl-siRNA = 3.34, siRNA-1 = 2.32, siRNA-2 = 2.61), *COL1A1* (fold change with TGF-β1; Ctrl-siRNA = 2.02, siRNA-1 = 1.44, siRNA-2 = 1.43), and *FN1* (fold change with TGF-β1; Ctrl-siRNA = 3.04, siRNA-1 = 2.15, siRNA-2 = 2.27) (Fig. 3B–E). Similarly, immunoblot analyses (Fig. 3F) and immunocytochemistry (Fig. 3G) confirmed the significant role of SNAIL in inducing the protein expression of α-SMA, SM22, type I collagen, and fibronectin following TGF-β1 stimulation to MIO-M1 cells. These results revealed that Müller glial cells are equipped with the Type 2 EMT multi-step cascade from pro-fibrotic TGF-β1 signaling to SNAIL activation and resultant cellular phenotypes, *i*.*e*., cytoskeleton contractility and ECM productivity.

### SNAIL is required for TGF-β1-induced cell motility in Müller glial cells

In addition to SNAIL-mediated expression of EMT-related molecular markers (Fig. 3), we next investigated whether SNAIL is required for TGF-β1-induced cell motility, an indispensable cellular feature for EMT, in Müller glial cells. *SNAI1* knockdown in MIO-M1 cells significantly suppressed TGF-β1-induced cellular activities including cell migration (ratio with TGF-β1; Ctrl-siRNA = 2.43, siRNA-1 = 1.57, siRNA-2 = 1.67) (Fig. 4A,B), cell invasion (ratio with TGF-β1; Ctrl-siRNA = 2.08, siRNA-1 = 1.50, siRNA-2 = 1.27) (Fig. 4C,D), and cell proliferation (ratio with TGF-β1; Ctrl-siRNA = 1.43, siRNA-1 = 1.09, siRNA-2 = 1.10) (Fig. 4E). These results showing that SNAIL contributes to TGF-β1-induced MIO-M1 cell motility, in concert with data on cytoskeleton contractility and ECM productivity (Fig. 3), suggested that the TGF-β1-SNAIL axis fully promotes Type 2 EMT cellular phenotypes in Müller glial cells.

### SNAIL is required for TGF-β1-induced loss of Müller glial property

Next, we investigated whether the TGF-β1-SNAIL axis affects the Müller glial property of MIO-M1 cells, with reference to the expression of glutamine synthetase (GS), encoded by the *GLUL* gene, which is widely used as a specific Müller glial marker^13,21,22^. TGF-β1 application to MIO-M1 cells significantly suppressed *GLUL* gene expression (fold change; 6 h = 0.70, 12 h = 0.58, 24 h = 0.66, 48 h = 0.71, 72 h = 0.65) and GS protein levels in a time-dependent manner (Fig. 5A,B). Importantly, *SNAI1* knockdown significantly recovered TGF-β1-induced decreases in the glial marker expression at both mRNA levels (fold change with TGF-β1; Ctrl-siRNA = 0.56, siRNA-1 = 0.88, siRNA-2 = 0.82) (Fig. 5C) and protein expression via immunoblot analyses (Fig. 5D) and immunocytochemistry (Fig. 5E). These results showing that Müller cells lose their glial property via the TGF-β1-SNAIL axis, in concert with myofibroblastic phenotypes obtained under the Type 2 EMT program (Figs 2–4), led us to propose the nomenclature “Müller GMT”, which should be distinctly discriminated from Type 3 EMT (carcinogenesis)-mimicking GMT in glioblastoma cells of brain astroglial origin^23–25^.

### SNAIL co-localizes with TGF-β ligand-receptor system in cells positive for myofibroblastic and Müller glial markers in iERM patient specimens

**Figure 6:**
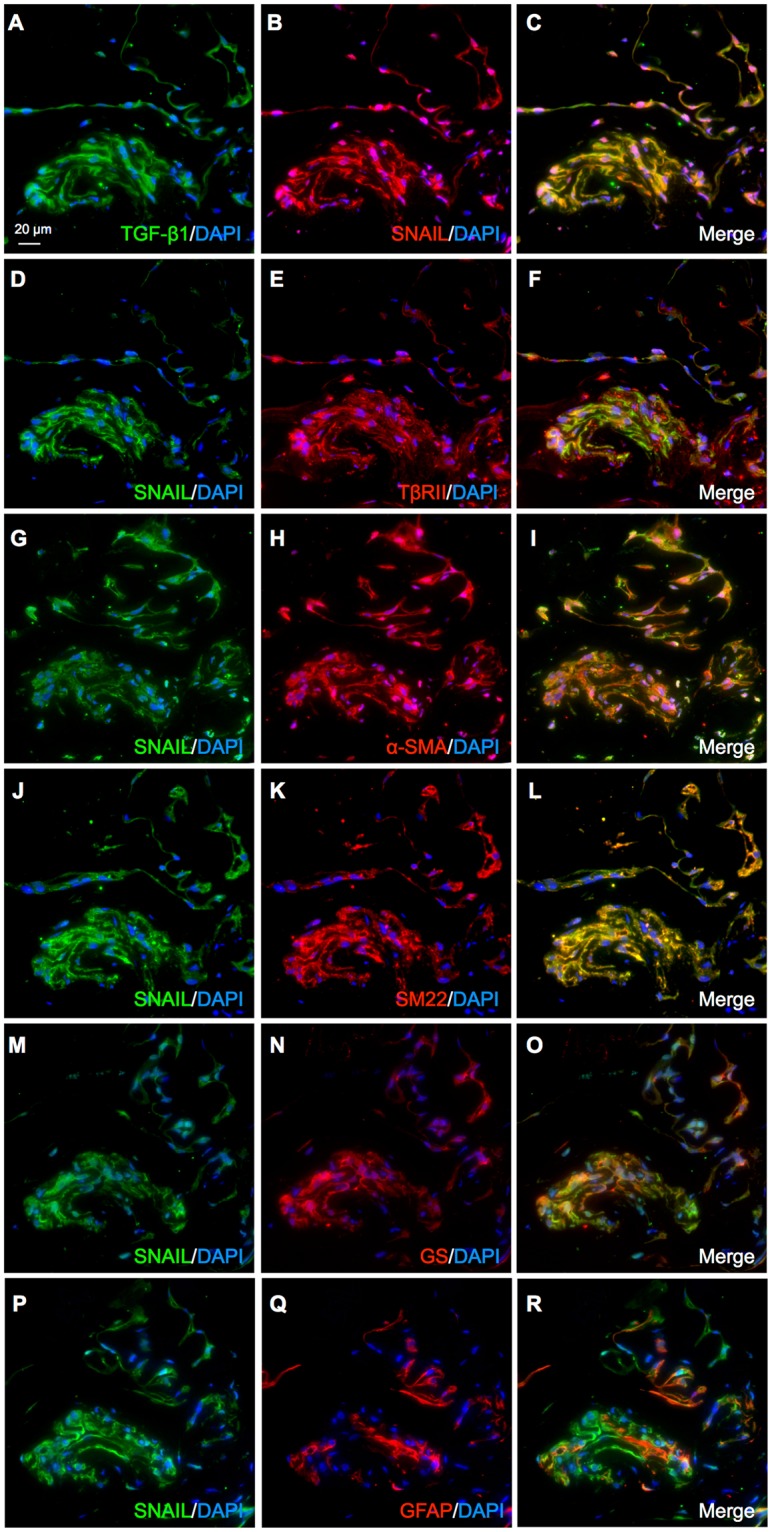
SNAIL co-localizes with TGF-β ligand-receptor system in cells positive for myofibroblastic and Müller glial markers in iERM patient specimens. (**A–O**) Double labeling of TGF-β1 (*green*) and SNAIL (*red*) (**A–C**), SNAIL (*green*) and TβRII (*red*) (**D–F**), SNAIL (*green*) and α-SMA (*red*) (**G–I**), SNAIL (*green*) and SM22 (*red*) (**J–L**), SNAIL (*green*) and GS (*red*) (**M–O**), and SNAIL (*green*) and GFAP (*red*) (**P–R**) in the iERM tissue specimens with DAPI (*blue*) counterstain to nuclei. Scale bar = 20 μm.

Because Müller GMT is driven by the TGF-β1-SNAIL axis in Müller glial cells with myofibroblastic differentiation (Figs 2–5), we analyzed related protein expression and localization via immunofluorescence in iERM tissues excised from patients during vitrectomy. Double staining experiments for serial sections demonstrated tissue co-localization of SNAIL with TGF-β1 (Fig. 6A–C), TβRII (Fig. 6D–F), α-SMA (Fig. 6G–I), SM22 (Fig. 6J–L), and GS (Fig. 6M–O), suggesting the activation of the SNAIL pathway via the TGF-β ligand-receptor system in myofibroblasts being transdifferentiated from Müller glial cells. Interestingly, SNAIL did not co-localize with glial fibrillary acidic protein (GFAP), a known astrocyte and Müller glial marker (Fig. 6P–R). Normal isotype IgGs showed no immunostaining (data not shown). These results demonstrated the potential establishment of Müller GMT in the pathogenesis of human iERM.

## Discussion

**Figure 7:**
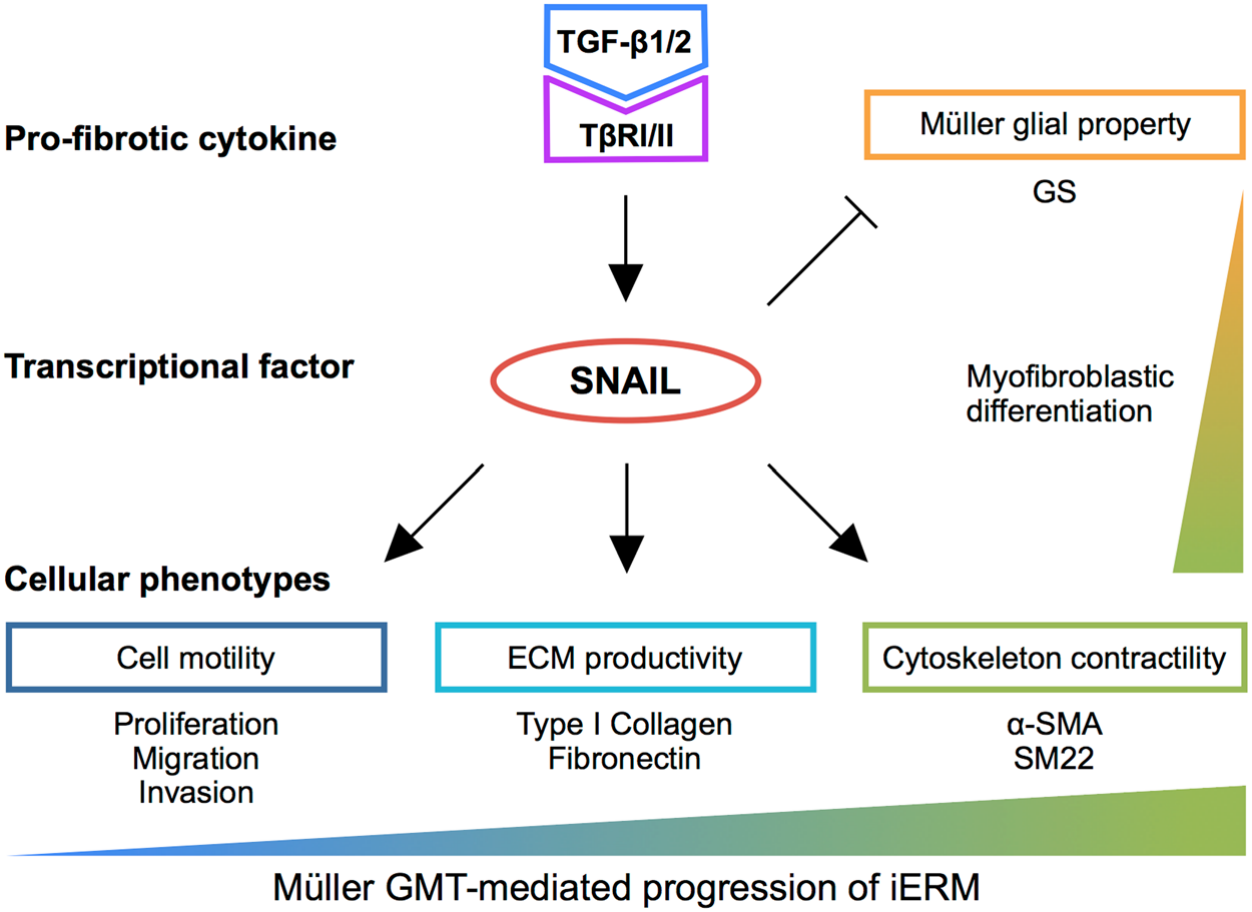
TGF-β-SNAIL axis induces Müller GMT in the pathogenesis of iERM. TGF-β-SNAIL axis promotes Type 2 EMT phenotypes, *i*.*e*., cell motility, ECM productivity, and cytoskeleton contractility, while attenuating glial property in Müller cells with myofibroblastic differentiation.

Although fibrogenic processes estimated from iERM tissue samples^10–12^ appear to be equivalent with Type 2 EMT featuring myofibroblasts^1,4,5^, the precise mechanism of myofibroblastic differentiation remains largely elusive in the pathogenesis of iERM that lacks epithelial contribution in nature. The present study demonstrated, for the first time to our knowledge, that Müller glial cells are equipped with the Type 2 EMT program driven by the TGF-β-SNAIL axis. Of various pro-fibrotic cytokines examined, TGF-β alone exclusively induced the expression of EMT markers in Müller glial cells (Fig. 1). As for transcription factors, TGF-β1 upregulated SNAIL, but not SLUG or TWIST, prior to the expression of myofibroblastic markers (α-SMA and SM22) and ECM proteins (type I collagen and fibronectin) in Müller glial cells (Fig. 2). Importantly, *SNAI1* knockdown in TGF-β1-stimulated Müller glial cells revealed the significant role of SNAIL in acquiring Type 2 EMT phenotypes, *i*.*e*., cytoskeleton contractility, ECM productivity (Fig. 3), and cell motility and growth (Fig. 4), together with loss of Müller glial property (Fig. 5). These *in vitro* data were further supported by immunohistochemistry for iERM patient specimens showing the tissue co-localization of SNAIL with TGF-β ligand-receptor system in GS-positive Müller glial cells undergoing myofibroblastic differentiation with α-SMA and SM22 expression (Fig. 6). These results led us to propose the pathological concept of Müller GMT, as an alternative to EMT, in the fibrogenic activity of iERM. The establishment of Müller GMT in the pathogenesis of iERM proved to be based on the specific combination of the pro-fibrotic cytokine TGF-β and the transcription factor SNAIL, together with resultant cellular phenotypes for myofibroblastic differentiation (Fig. 7).

The nomenclature GMT was first described in a recent oncologic report showing irradiation-induced mesenchymal (EMT-like) changes in malignant glioma cells of brain astrocytic (*i*.*e*., non-epithelial) origin, which would reasonably explain its higher rate of recurrence with more aggressive phenotypes after treatment^23^. Subsequent reports also confirmed that the carcinogenic Type 3 EMT program originally used in epithelial tumor cells would be appropriated to glioblastoma cells with SNAIL activation due to irradiation, hypoxia, and TGF-β^24,25^. Glial cells are defined as non-neuronal and non-vascular cells present in the central nervous system, and retinal glial cells consist of Müller cells, astrocytes, and microglial cells (retinal resident macrophages), all of which are quite different in function, distribution, structure, and origin (retina, brain, and bone marrow, respectively)^21^. Müller GMT with the Type 2 EMT program featuring myofibroblasts should therefore be distinguished from GMT in motile tumor cells of astroglial origin mimicking Type 3 EMT (hereafter referred to as “astroGMT” or astroglial-mesenchymal transition).

The clinical entity of iERM was previously termed epiretinal fibrosis or epiretinal gliosis, the latter of which represents reactive Müller cell gliosis (Müller gliosis) marked by the upregulation of intermediate filaments such as vimentin and GFAP (Supplementary Fig. S4A–C)^14,15^. Brain injury basically causes reactive astrogliosis characterized by the dense meshwork of GFAP-enriched cytoplasmic processes of fibrillary astrocytes (*i*.*e*., glial fibrils), a hallmark of glial scar devoid of ECM deposition^26^. These cellular reactions, whether astrogliosis or Müller gliosis, stand in contrast to fibrosis as well as fibrotic scar consisting of collagen fibrils (ECM deposition) secreted by myofibroblasts. Indeed, fibrogenic Müller GMT was associated with unchanged vimentin (Fig. 2F) and decreased GFAP (Supplementary Fig. S4D) expression, underscoring an inconsistency between Müller GMT and Müller gliosis in the direction of cell differentiation, *i*.*e*., losing and enhancing glial property, respectively. Co-existence of fibrotic and gliotic Müller cells during iERM formation, if only the timing of differentiation is deviated, appears to be specific to the eye, because brain astrocytes are thought to have no potential for or involvement in fibrosis^26,27^. Consistently, the recently identified astroGMT is related with tumor progression (Type 3 EMT) but not with fibrosis (Type 2 EMT)^23–25^.

In ocular fibrosis, TGF-β has long been suggested to induce myofibroblastic differentiation not only in cells of epithelial origin such as RPE cells^5,6^ but in Müller glial cells^10,11^. In the epiretinal fibrous tissue associated with iERM and idiopathic macular hole, Müller cells were suggested to migrate, proliferate, and transdifferentiate into myofibroblasts^10–13,28^; however, the precise mechanism of myofibroblastic differentiation driven by the Type 2 EMT program was not fully documented. The currently observed TGF-β-SNAIL axis in Müller glial cells was also shown to apply to RPE cells as the *bona fide* Type 2 EMT program to promote subretinal fibrosis in neovascular age-related macular degeneration^6,9^. Müller glial cells were also shown to be involved with proliferative diabetic retinopathy complicated by epiretinal fibrovascular growth, in terms of facilitating both angiogenesis^22,29^ and fibrogenesis^30^. Notably, high glucose stimulation to Müller cells was shown to induce SNAIL-mediated CTGF and fibronectin expression^30^, which would imply another example of Müller GMT involved in the fibrogenic activity of proliferative diabetic retinopathy.

The present study clearly demonstrated the significance of Müller GMT as the precise mechanism of myofibroblastic differentiation from non-epithelial cells; however, other cellular participants could not definitely be denied as the origin of myofibroblasts during the fibrogenic processes of iERM. Hyalocytes (vitreal resident macrophages) are bone marrow-derived cells of monocytic origin and normally present in the posterior vitreous cortex, a potential scaffold for fibrocellular proliferation in iERM^10^. Importantly, TGF-β2 stimulation to hyalocytes was shown to induce α-SMA expression and enhance collagen gel contraction^31^, suggesting the possibility of the Type 2 EMT program equipped in this cell type. In parallel, astrocytes exhibited only marginal changes in α-SMA expression and contractile activity in response to TGF-β2^31^. Astrocytes express GFAP more predominantly than Müller glial cells^10^, together with a neural tissue marker S100^32^ as shown in our iERM samples (Supplementary Fig. S4E–G). GFAP-positive and GS-negative astrocytes in the iERM tissue demonstrated no positive staining for α-SMA^31^, suggesting the unlikeliness of astroGMT as a driving force of myofibroblastic differentiation. This observation is supported by ours showing tissue co-localization of SNAIL with GS (Fig. 6M–O) but not GFAP (Fig. 6P–R) in iERM patient specimens. The presence of astrocytes has been confirmed, however, with ultrastructural and immunohistochemical approaches in various fibrotic disorders associated with macular hole retinal detachment^33^ and cavitary optic disc anomalies^32^, warranting future research to clarify the role of astrocytes in epiretinal fibrous growth. Fibrocytes are bone marrow-derived, circulating blood cells with mixed characteristics of monocytes and fibroblasts, and infiltrate into various fibrogenic lesions with myofibroblastic differentiation^34^. The association of fibrocytes with ocular fibrosis was not reported in iERM but in more inflammatory retinopathies such as proliferative vitreoretinopathy^35^ and proliferative diabetic retinopathy^36,37^. Vascular cells, *i*.*e*., pericytes and endothelial cells, both possess a potential to transdifferentiate into myofibroblasts, and the fibrovascular tissue collected from patients with proliferative diabetic retinopathy showed myofibroblastic differentiation from vascular endothelial cells (commonly known as “EndoMT” or endothelial-mesenchymal transition)^36^. The involvement of vascular cells in ocular fibrosis would thus be more likely in neovascular diseases than in iERM characterized by fibrocellular proliferation lacking vascular components.

In conclusion, this study highlights the novel concept of Müller GMT in the pathogenesis of iERM, showing that Müller glial cells are equipped with the SNAIL-mediated Type 2 EMT program. Müller GMT is established on a basis of the specific combination of the pro-fibrotic cytokine TGF-β and the transcription factor SNAIL, together with resultant cellular phenotypes for myofibroblastic differentiation. Our present data shed light on further understanding of the molecular pathogenesis of ocular fibrosis, so as to allow future development of new pharmacologic therapies for the prevention of vision loss.

## Methods

### Cell line and reagents

The human Müller glial cell line Moorfields/Institute of Ophthalmology-Müller 1 (MIO-M1) was provided from Dr. G. Astrid Limb (UCL Institute of Ophthalmology, London, United Kingdom)^38^. The cells were cultured in DMEM containing 10% fetal bovine serum (FBS) (Thermo Fisher Scientific, Waltham, MA).

Recombinant human TGF-β1, TGF-β2, BMP-4, CTGF, GDNF, NGF, FGF2, and PDGF-BB proteins were purchased from PeproTech (Rocky Hill, NJ) and used at 10 ng/ml for 24 hours. Mouse anti-TGF-β1 neutralizing antibody and normal mouse IgG were purchased from R&D Systems (Minneapolis, MN) and preincubated at the dose of 200 ng/ml for 15 minutes with 10 ng/ml TGF-β1.

Two siRNA oligos for suppressing the gene expression of *SNAI1* (siRNA-1, hs.Ri.SNAI1.13.1; siRNA-2, hs.Ri.SNAI1.13.2) and a negative control siRNA oligo (Ctrl-siRNA, DS NC1) were purchased from Integrated DNA Technologies (Coralville, IA) and used at 10 nM. Cells were transfected with siRNA using Lipofectamine RNAiMAX Reagent (Thermo Fisher Scientific) following the manufacturer’s protocols. Twenty-four hours after transfection, the composite transfection mixture was removed and replaced with 1% FBS/DMEM for 24 hours, followed by administration with recombinant human TGF-β1 before each assay.

### Real-time quantitative PCR

Total RNA isolation, DNase I treatment, and reverse transcription were performed from cells using SuperPrep Cell Lysis & RT Kit for qPCR (TOYOBO, Tokyo, Japan) following the manufacturer’s protocols. All the primer sequences used in the current study are listed in Table 1. Real-time quantitative PCR was performed using the GoTaq qPCR Master mix (Promega, Madison, WI) and StepOne Plus Systems (Thermo Fisher Scientific). Gene expression levels were calculated using the 2^−ddCt^ method. The correct amplification of a specific product was confirmed by the dissociation temperature of the product and agarose gel electrophoresis. All experimental samples were normalized using human *GAPDH* as an internal control.

**Table 1:** Primer sequences used in real-time quantitative PCR.

Target gene	Sequence
*ACTA2*	forward 5′- TCT GTA AGG CCG GCT TTG C -3′
	reverse 5′- TGT CCCA TTC CCA CCA TCA -3′
*COL1A1*	forward 5′- GAG GGC CAA GAC GAA GAC ATC -3′
	reverse 5′- CAG ATC ACG TCA TCG CAC AAC -3′
*FN1*	forward 5′- CAA GTAT GAG AAG CCT GGG TCT -3′
	reverse 5′- TGA AGA TTG GGG TGT GGA AG -3′
*GFAP*	forward 5′- CCT CTC CCT GGC TCG AAT G -3′
	forward 5′- GGA AGC GAA CCT TCT CGA TGT A -3′
*LAMA1*	forward 5′- AAT GGT GCT GGC AGG ATA AC -3′
	reverse 5′- CCA GGA CAG GAA TGA AGG AA -3′
*SNAI1*	forward 5′- TCG GAA GCC TAA CTA CAG CGA -3′
	reverse 5′- AGA TGA GCA TTG GCA GCG AG -3′
*SNAI2*	forward 5′- CGA ACT GGA CAC ACA TAC AGT G -3′
	reverse 5′- CTG AGG ATC TCT GGT TGT GGT -3′
*TAGLN*	forward 5′- TCC AGA CTG TTG ACC TCT TTG -3′
	reverse 5′- CAT CAT CCT CAC TGC TTC TGT 3′
*TWIST1*	forward 5′- CGG GAG TCC GCA GTCTTA -3′
	reverse 5′- GCT TGA GGG TCT GAA TCT TG -3′
*VIM*	forward 5′- GAC GCC ATC AAC ACC GAG TT -3′
	reverse 5′- CTT TGT CGT TGG TTA GCT GGT -3′
*GAPDH*	forward 5′- CCT GGC CAA GGT CAT CCA TG -3′
	reverse 5′- GGA AGG CCA TGC CAG TGA GC -3′

### Immunoblot analyses

Cell extracts were lysed in SDS buffer and a protease inhibitor cocktail (Promega). After quantifying protein concentrations using BCA reagent (Thermo Fisher Scientific), proteins were resolved by SDS-PAGE (polyacrylamide gel electrophoresis) and transferred to PVDF membrane by electroblotting. Membranes were blocked in TBS containing 5% skim milk, and probed with the following primary antibodies: mouse anti-α-SMA (1:2000, R&D Systems), rabbit anti-SM22 (1:2000), rabbit anti-type I collagen (1:2000), mouse anti-fibronectin (1:2000), mouse anti-glyceraldehyde-3-phosphate dehydrogenase (GAPDH) (1:2000, Thermo Fisher Scientific), rabbit anti-SNAIL (1:1000, Cell signaling technology, Danvers, MA), and mouse anti-GS (1:1000, Millipore, Temecula, CA) antibodies. Horseradish peroxidase-conjugated anti-mouse and anti-rabbit IgGs (1:4000, Jackson ImmunoResearch Laboratories, West Grove, PA) were used as secondary antibodies for chemoluminescence detection. Signals were obtained by enhanced chemoluminescence (Perkin Elmer, Waltham, MA).

### Immunocytochemistry

MIO-M1 cells were cultured in 8-well multichamber slides (Thermo Fisher Scientific). The cells were washed with PBS, permeabilized with ice-cold methanol for 20 min, and washed again in PBS. After blocking with 5% goat serum, the cells were incubated with the following primary antibodies: mouse anti-α-SMA (1:200, R&D Systems), rabbit anti-SM22 (1:200), rabbit anti-type I collagen (1:200), mouse anti-fibronectin (1:200, Thermo Fisher Scientific), and mouse anti-GS (1:100, Millipore) antibodies. Secondary antibodies for fluorescent detection were AlexaFluor 488 and 546 (1:400, Thermo Fisher Scientific). Nuclei were counterstained with DAPI (4′,6-diamidino-2-phenylindole), and cells were examined using a Biorevo BZ-9000 microscope (Keyence, Osaka, Japan).

### Cell migration assay

MIO-M1 cell migration was evaluated using an Oris 96-well cell migration assay kit (Platypus Technologies, Madison, WI), according to the manufacturer’s instructions. Cells were seeded in each well and transfected with siRNAs. After 1 hour of pretreatment with 1% FBS/DMEM containing 10 ng/ml TGF-β1 with 5 mM aphidicolin (Fuji Film Wako Pure Chemicals, Osaka, Japan) to inhibit cell division, the stoppers were removed to allow cells to migrate into the detection zone. The cells were then incubated for 48 hours and stained with PBS containing calcein AM (Dojindo, Kumamoto, Japan) for 1 hour. The signal intensity of the stained cells that migrated into the detection zone was measured with an Infinite F200 PRO microplate reader in the fluorescence mode (TECAN, Männedorf, Switzerland) using a fluorescence filter set (excitation, 485 nm; emission, 535 nm) with an Oris detection mask attached to the bottom of the plate.

### Cell invasion assay

MIO-M1 cell invasion was assessed using a BioCoat Matrigel Invasion Chamber (Corning, Bedford, MA), according to the manufacturer’s instructions. Chambers were allowed to defrost at room temperature and rehydrated in DMEM. siRNA-transfected cells were then seeded in the upper well, while 10 ng/ml TGF-β1 containing 1% FBS/DMEM was applied in the lower well, followed by incubation for 24 hours. The remaining cells in the upper well were removed by wiping. The cells moved to the lower side of the filter were fixed with 100% methanol and stained with Toluidine Blue O (Fuji Film Wako Pure Chemicals). The total number of invading cells was quantified by counting in 5 random fields.

### Cell proliferation assay

MIO-M1 cell proliferation was quantified using BrdU (5-bromo-2′-deoxyuridine) ELISA (Roche Applied Science, Indianapolis, IN), according to the manufacturer’s instructions. In brief, siRNA-transfected cells were stimulated with 10 ng/ml TGF-β1 containing 1% FBS/DMEM for 24 hours, and incubated with BrdU for 2 hours. The optical density was determined using a microplate reader (Sunrise, TECAN).

### Human surgical samples

iERM tissues were surgically excised from 6 eyes of 6 patients during pars plana vitrectomy, and used for immunofluorescence analyses. The clinical characteristics of the patients in this study are listed in Table 2. This study was conducted in accordance with the tenets of the Declaration of Helsinki and after receiving approval from the institutional review board of Hokkaido University Hospital. All patients gave written informed consent after our explanation of the purpose and procedures of this study (IRB #015-0226).

**Table 2:** Clinical characteristics of patients with iERM.

Case	Age (years)	R/L	Sex	History of ocular surgery	Decimal visual acuity
1	75	L	F	None	0.4
2	62	R	F	None	0.4
3	75	R	M	Cataract (5 years ago)	0.6
4	76	L	F	None	0.9
5	70	R	M	None	0.5
6	52	L	F	None	0.5

### Immunofluorescence microscopy

Immunofluorescence analyses were performed as described previously^12^. Paraffin sections of iERM tissues were deparaffinized and hydrated through exposure with xylene and graded alcohols followed by water. As a pretreatment, microwave-based antigen retrieval was performed in 10 mM citrate buffer (pH 6). Sections were incubated with the following primary antibodies: rabbit anti-SNAIL (1:100, Proteintech, Rosemont, IL), mouse anti-TGF-β1 (1:50), mouse anti-S100 (1:50, Thermo Fisher Scientific), goat anti-TβRII (1:50), mouse anti-α-SMA (1:200, R&D Systems), goat anti-SM22 (1:100, Abcam, Cambridge, UK), mouse anti-GS (1:100, Millipore), and mouse anti-GFAP (1:100, Leica, Exton, PA) antibodies. Secondary antibodies for fluorescent detection were AlexaFluor 488 and 546 (1:400, Thermo Fisher Scientific). Nuclei were counterstained with DAPI, and sections were examined using a Biorevo BZ-9000 microscope (Keyence).

### Statistical analyses

All the results are expressed as the mean ± SEM (standard error of the mean). Student’s t-test was used for statistical comparison between groups, and one-way analysis of variance (ANOVA) followed by the Tukey-Kramer method as a post-hoc test was used for multiple comparison procedures. Differences between means were considered statistically significant when *p* values were < 0.05.

## Supplementary information

Supplementary figures
