# Supplementary material for: TGF-β-SNAIL axis induces Müller glial-mesenchymal transition in the pathogenesis of idiopathic epiretinal membrane
Source: Sci Rep. 2019 Jan 24;9:673. doi: 10.1038/s41598-018-36917-9 (PMC6346093; doi:10.1038/s41598-018-36917-9)
Supplement: Supplementary file 1 — Supplementary figures [file 41598_2018_36917_MOESM1_ESM.pdf]

TGF-β-SNAIL axis induces Müller glial-mesenchymal transition in the pathogenesis of idiopathic epiretinal membrane

Atsuhiro Kanda, Kousuke Noda, Ikuyo Hirose, Susumu Ishida

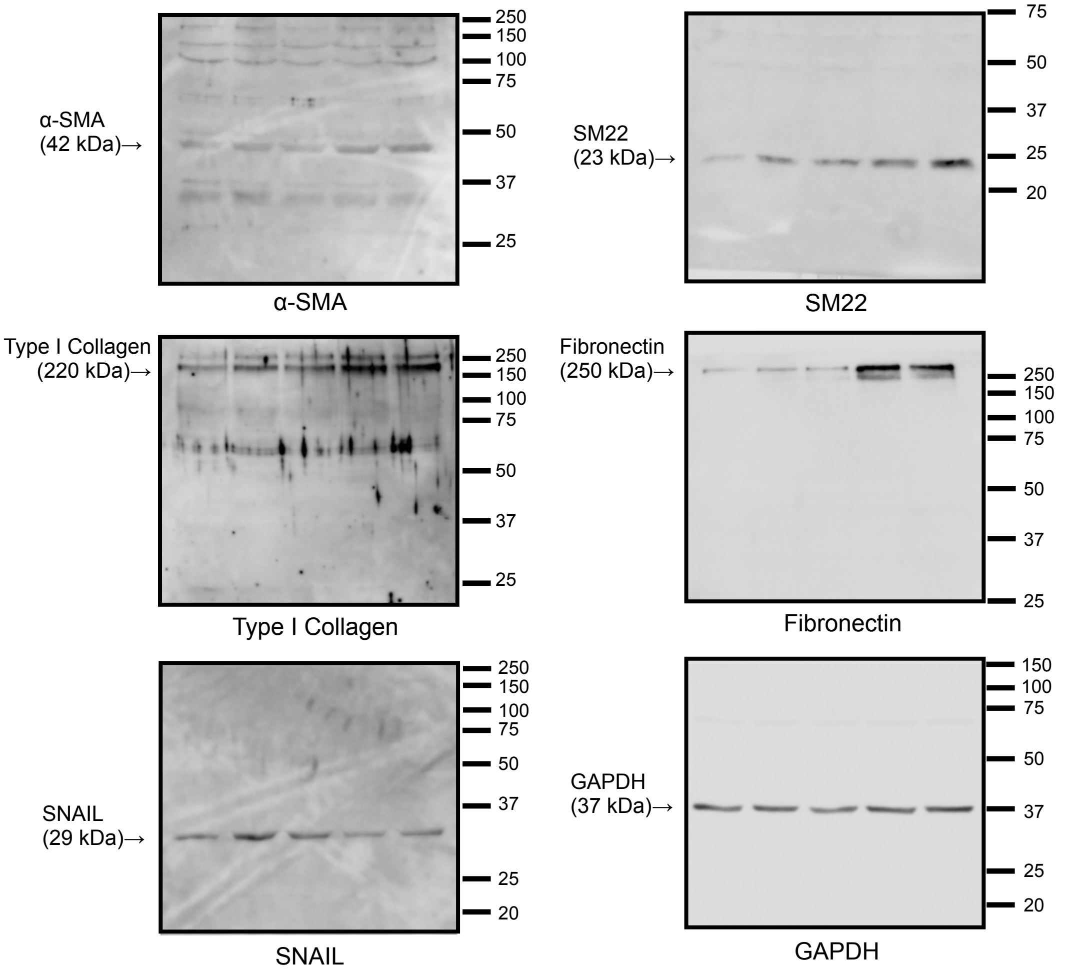

Supplementary Figure S1. The full length blots of Fig. 2J.

TGF-β-SNAIL axis induces Müller glial-mesenchymal transition in the pathogenesis of idiopathic epiretinal membrane

Atsuhiro Kanda, Kousuke Noda, Ikuyo Hirose, Susumu Ishida

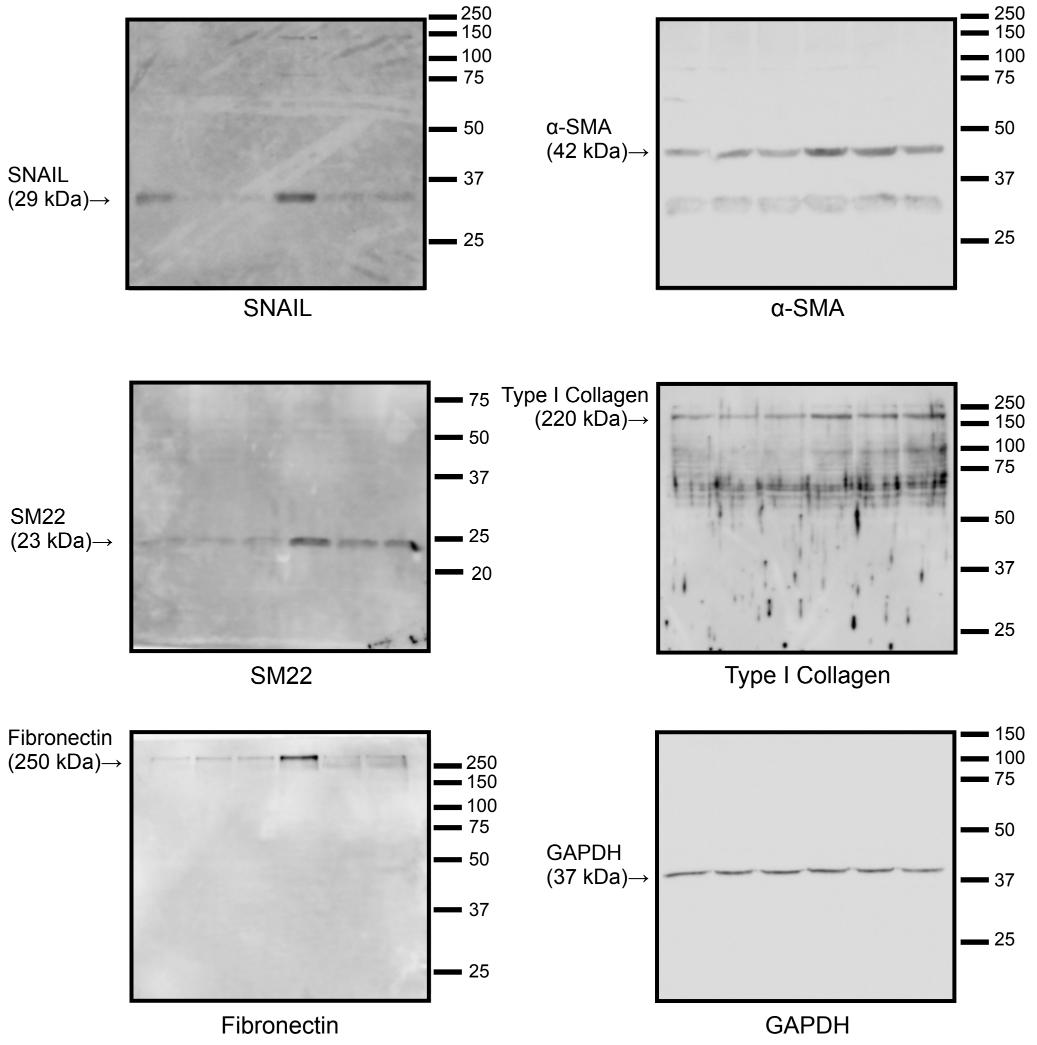

Supplementary Figure S2. The full length blots of Fig. 3F.

TGF-β-SNAIL axis induces Müller glial-mesenchymal transition in the pathogenesis of idiopathic epiretinal membrane

Atsuhiro Kanda, Kousuke Noda, Ikuyo Hirose, Susumu Ishida

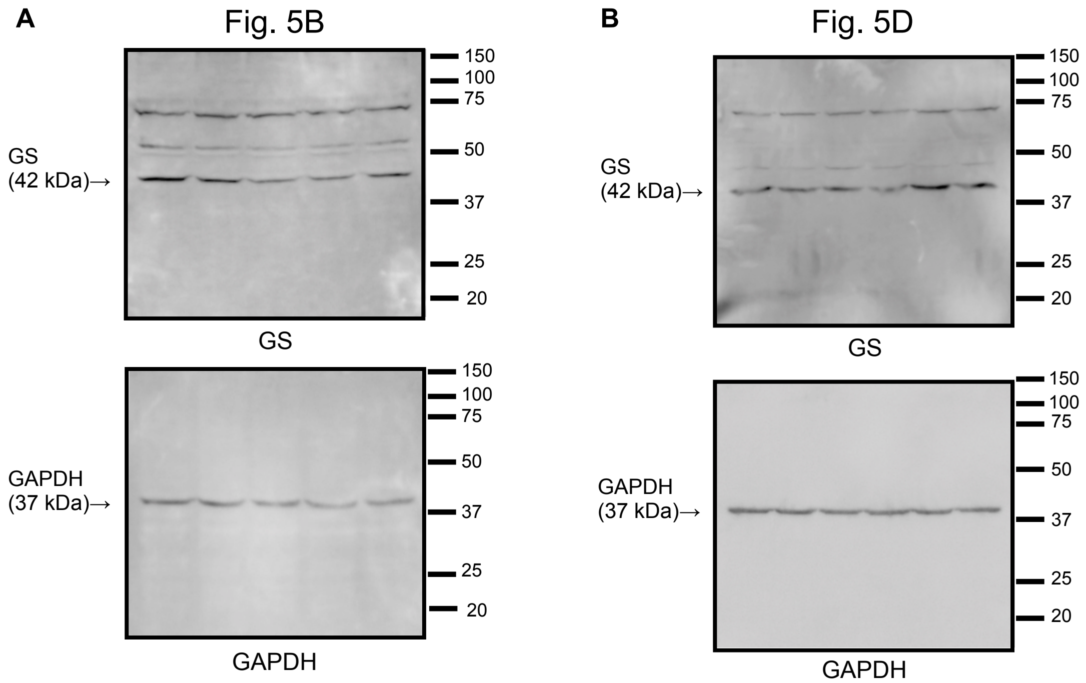

Supplementary Figure S3. The full length blots of Fig. 5B and D.

TGF-β-SNAIL axis induces Müller glial-mesenchymal transition in the pathogenesis of idiopathic epiretinal membrane

Atsuhiro Kanda, Kousuke Noda, Ikuyo Hirose, Susumu Ishida

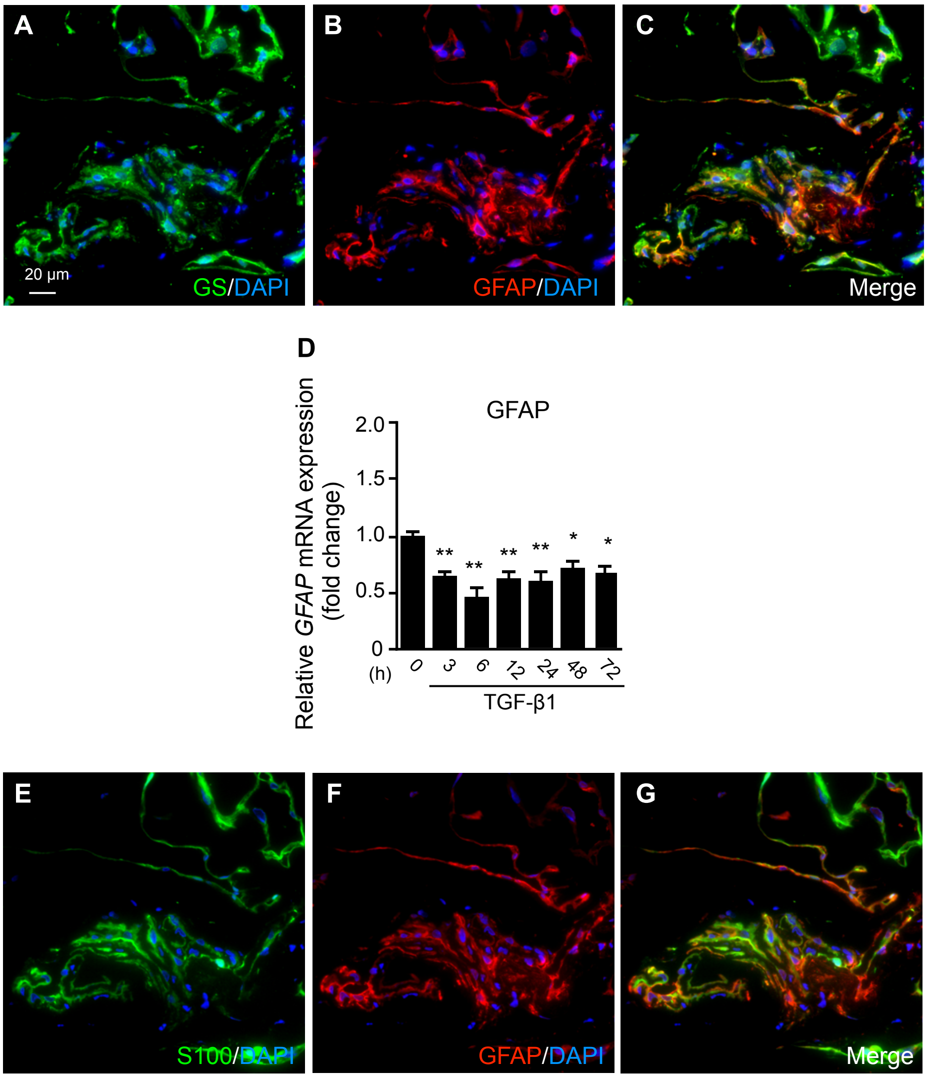

Supplementary Figure S4. Localization and expression analyses of GFAP in iERM patient specimens and Müller glial cells. (**A-C**) Double labeling of GS (*green*) and GFAP (*red*) in the iERM tissue specimens with DAPI (*blue*) counterstain to nuclei. Scale bar = 20 µm. (**D**) Real-time quantitative PCR analyses for the time course of expression levels of *GFAP*. h, hours. n = 6 per group, $*p < 0.05$, $**p < 0.01$. (**E-G**) Double labeling of S100 (*green*) and GFAP (*red*) in the iERM tissue specimens with DAPI (*blue*) counterstain to nuclei.
